# Stress shielding effects of two prosthetic groups after total hip joint simulation replacement

**DOI:** 10.1186/s13018-014-0071-x

**Published:** 2014-08-30

**Authors:** Chengdong Piao, Dankai Wu, Min Luo, Hongshun Ma

**Affiliations:** 1 Department of Orthopaedics, The Second Hospital of Jilin University, Changchun 130041, Jilin Province, China; 2 Department of Orthopedics, China-Japan Union Hospital, Jilin University, Changchun 130031, Jilin Province, China; 3 Department of Engineering Mechanics, College of Mechanical Science and Engineering, Jilin University, Changchun 130022, Jilin Province, China; 4 Department of Orthopaedics, China-Japan Friendship Hospital, Jilin University, Changchun 130031, Jilin Province, China

**Keywords:** Hip joint, Artificial prosthesis, Replacement, Electric measurement

## Abstract

**Objective:**

The study aims to compare the stress shielding effects of implantable anatomical and traditional prostheses after *in vitro* total hip joint replacement simulation. The study serves as a biomechanical basis for novel artificial prostheses and for clinical hip joint replacements.

**Methods:**

Sixteen femoral specimens from adult male corpses were randomly divided into two groups: the traditional prosthesis group implanted into femur specimens using simulated total hip joint replacement (*n* = 8) and the femoral neck-preserved anatomical prosthesis implantation group that used a collum femoris preserving stem/trabeculae oriented pattern (CFP/TOP) acetabular cup (*n* = 8). The strain values in the two groups before and after prosthesis implantation were measured at different test points using electric resistance strain gauges. The stress shielding rate was calculated according to the related formula.

**Results:**

The results showed that the rates of proximal femoral stress shielding were significantly higher at test points 1–10 in the traditional femoral prosthesis transplantation group than in the anatomical prosthesis group (*p* < 0.05).

**Conclusions:**

There were different effects of stress shielding between the anatomical and traditional prostheses. Retained femoral anatomical implants should reduce stress shielding and increase the stability of anatomical prosthesis implants.

## Introduction

Although total hip joint simulation replacement has been considered to be the most successful and influential orthopedic surgery of the twentieth century, short prosthesis life is its fatal flaw. Important factors, including the prosthesis design, installation, materials, and others, can influence the lifespan of the prosthesis [1]. Femur bone remodeling and bone loss continue after hip replacement surgery, especially in the proximal femur. After replacement, the stress shielding of the proximal femur is considered to be the mechanical cause of bone loss. Bone loss and cortical thinning eventually lead to joint prosthesis failure [2]. Studies on the stress shielding effect, after prosthesis implantation, have been receiving increased attention. Globally, researchers have studied the biomechanics of the hip joint and the stress shielding effects of artificial prostheses [3]–[7]. The results of an *in vitro* biomechanical experiment [8] confirmed that the lateral alar process of the stem reduces the stress shielding of the medial proximal femur and greatly increases the rotational stability of the stem in the medial bone cement sheath. Using the finite element method, Fouad [9] suggested that a subtle change in the artificial femoral head material can influence hip joint stress distribution; this phenomenon is mainly related to the elastic modulus of the artificial femoral head material. Beulah et al. [10] assessed a novel, low-elastic modulus prosthesis with a hexagonal cross-sectional design, using the finite element method and found that it is more effective in reducing stress shielding and in strengthening prosthesis fixation. Davis et al. [11] established a finite element analysis model, after reconstruction and replacement with a metal hip joint surface, and simulated the force to calculate stress values. After reconstruction and replacement with a metal hip joint surface, the stress was mainly concentrated inside the femoral neck and at the junction of the prosthesis and neck head; stress shielding occurred at the lower bony part of the femoral prosthesis.

Studies on the stress shielding effect, after total hip joint simulation replacement, focused on implanting different prostheses, mostly using the three-dimensional finite element method [8]–[11]. Studies that compare the stress shielding effects of two types (anatomical and traditional) of artificial prostheses, based on electric strain gauge measurement values before and after total hip joint simulation replacement, are lacking. In biomechanical research, the use of a strainmeter is a measurement technique for studying the stress shielding effect, after the implantation of various prostheses by total hip joint replacement simulation.

This study simulated total hip arthroplasty using normal, adult cadaver femurs. The measurement of electric strain was estimated before and after prosthesis placement using German retained prosthetic femoral neck-type and traditional prostheses. The strain, stress, and stress shielding rate values for each point were measured in the femur, under compressive stress, before and after placement of the prosthesis, comparing the retained femoral neck-type and the traditional prosthesis groups. We aimed to provide a biomechanical basis for novel artificial prostheses and clinical hip joint replacements by performing a quantitative comparative analysis of the effects of retained femoral neck prostheses and traditional prostheses implanted into femur specimens using electric strainmeter measurements.

## Materials and methods

### Specimens

A total of 16 femoral specimens from normal adult male corpses (age range, 20–30 years old; height range, 1.75–1.82 m; weight range, 74–85 kg (mean, 80 kg)) were provided by the Department of Anatomy, Bethune Medical University (Changchun, China). Within 2 h after death, the left and right femurs were removed, packed with normal saline-soaked gauze, placed into plastic bags, sealed, and stored at −20°C. This study was conducted in accordance with the declaration of Helsinki. This study was conducted with approval from the Ethics Committee of China-Japan Friendship Hospital, Jilin University.

### Grouping

**Figure 1:**
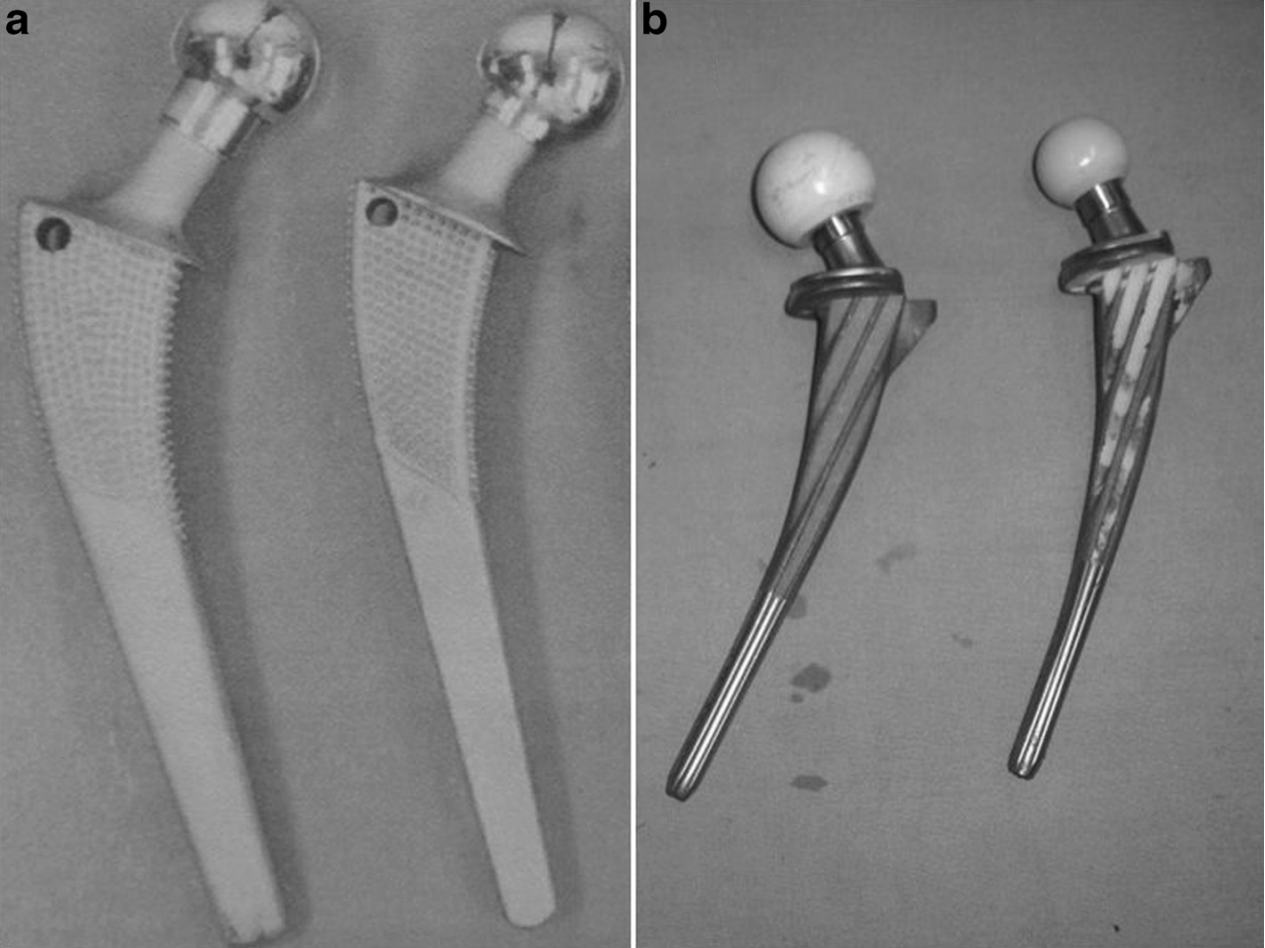
**The traditional prosthesis (a) and the femoral neck-preserved anatomical prosthesis (b).** (A) Length of the prosthetic handle is 150 mm, angle of prosthetic neck is 135°, and diameter of prosthetic head is 28 mm. (B) Length of prosthetic handle is 135 mm, angle of prosthetic neck is 126°, and diameter of prosthetic head is 28 mm.

**Figure 2:**
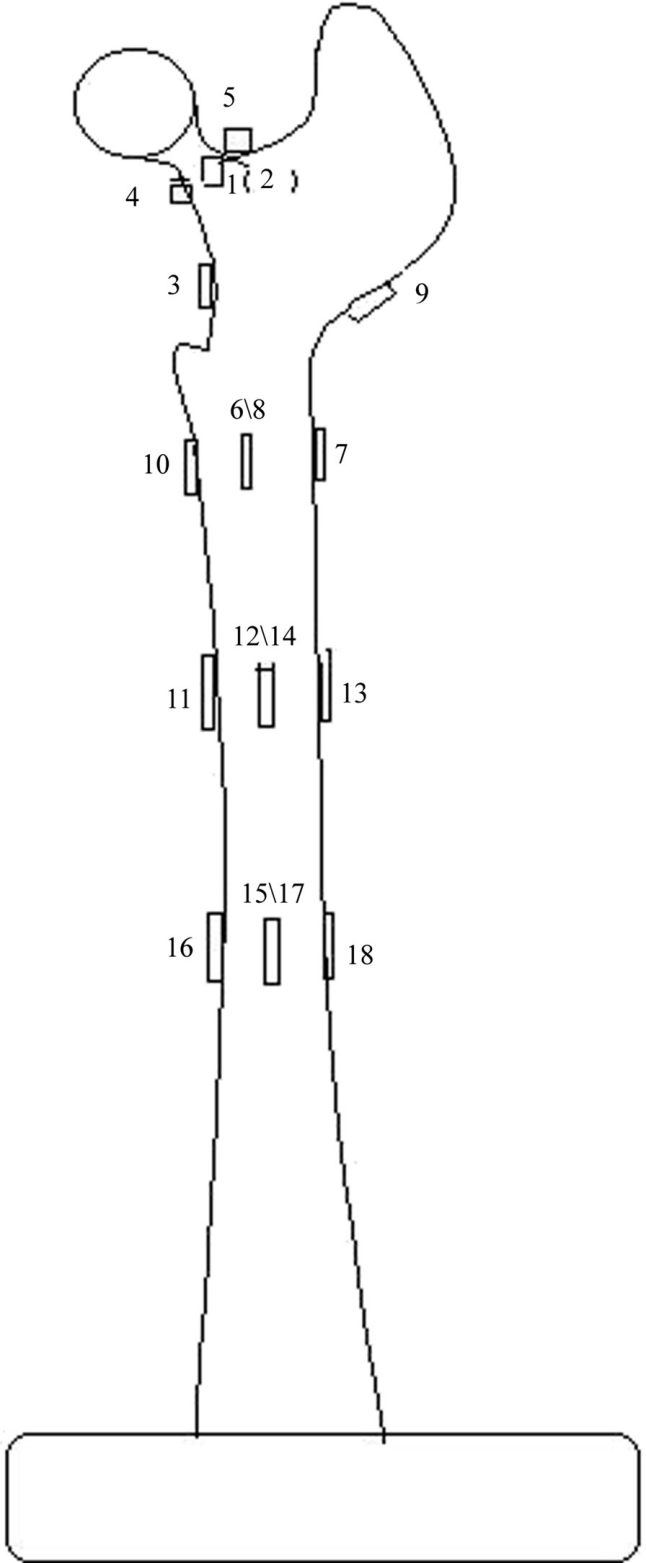
**Schematic of electric resistance strain gauge distribution of femoral specimens.** Measurement points 1, 2, 3, 4, and 5 were in the femoral neck area. Measurement point 4 was in the medial femoral neck at the upper 3 cm of the lesser trochanter; measurement point 3 was at the upper 1.5 cm of the lesser trochanter; measurement point 6 was at the transverse middle position of lesser trochanter; measurement point 8 was at the rear of measurement point 6; measurement point 7 was at the opposite side of the lower lesser trochanter; measurement point 9 was at the opposite side of the femoral neck; measurement point 10 was at the lower 1 cm of the lesser trochanter; measurement points 11, 12, 13, and 14 were stem tip positions of the anatomical prosthesis implantation group; and measurement points 15, 16, 17, and 18 were stem tip positions of the traditional prosthesis implantation group.

Before the experiment, the femoral specimens were thawed at room temperature. A total of 16 specimens (8 left and 8 right femurs) were randomly divided into two groups. In the traditional prosthesis group (*n* = 8), the prosthesis (Vatallium; Beijing Prussia Explant Material, Beijing, China) was implanted into the femur specimens using a total hip joint replacement simulation (Figure 1). In the femoral neck-preserved anatomical prosthesis group (*n* = 8), the collum femoris preserving stem/trabeculae oriented pattern (CFP/TOP) acetabular cup (titanium alloy; Waldemar LINK GmbH & Co. KG, Hamburg, Germany) was used (Figure 2).

### Femoral size measurement and embedding immobilization method

The length and diameter of the femoral specimens were measured using a steel ruler and a reading microscope, respectively. In the traditional femoral prosthesis group, the specimens were 366.4–428.8 mm long and 25.81–26.92 mm in diameter. In the anatomical prosthesis group, the femurs were 375.4–439.8 mm long and 25.82–27.32 mm in diameter. Before artificial prosthesis implantation, the femur specimens were 370.2–376.3 mm long and 25.81–27.32 mm in diameter. Leveling of the surface of the lower ends of the femurs was achieved by embedding immobilization, using diluted dental base acrylic resin powder at the lower end of each specimen. The lower end of each femur was placed in a mold, and the mold was filled with denture base resin powder and liquid (Shanghai New Century Dental Material, Shanghai, China) (powder/liquid ratio, 22 g:10 mL) to embed and fix the distal ends of the specimens.

### Electric measurements using strain gauges

**Figure 3:**
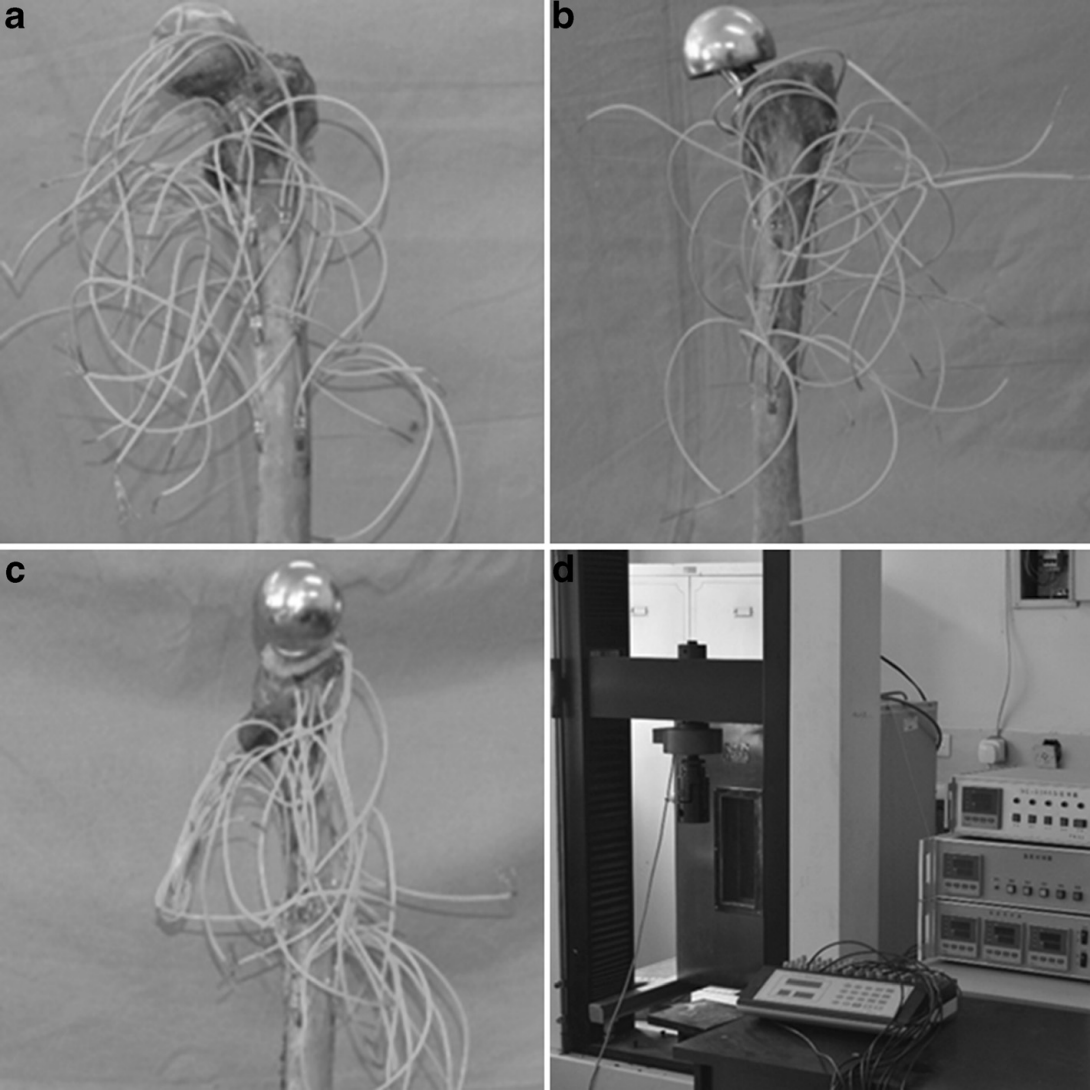
**Femoral electric strain measurement. (a)** Femoral electric strain measurement of the normal control group, **(b)** femoral electric strain measurement of the traditional prosthesis implantation group, **(c)** femoral electric strain measurement of the anatomical prosthesis implantation group, and **(d)** strain measurement was performed using electronic universal testing machine and imaged by resistance strain gauges.

To conduct electric measurements, gum-based, foil-type electric resistance strain gauges (Huangyan Testing Instrument Factory, Taizhou City, China) were affixed to the femurs in each group before prosthesis implantation in both groups. The length, resistance value, and sensitivity coefficient of the gauges were 2 × 2 mm, 120 ± 0.1 Ω, and 2.12, respectively. According to the requirements of the strain gauges, the adhesion positions were cleaned using alcohol and acetone before femoral prosthesis implantation. The strain gauges were affixed using Speedglue 502 (Beijing Chemical Works, Beijing, China) at various measurement points. Subsequently, the specimens were allowed to dry naturally for 24 h. A schematic of the electric resistance strain gauge placement at various femoral measurement points is shown in Figure 3.

### Strain electric measurements in pre-transplant normal femurs in the traditional and anatomical prosthesis implant groups

Before prosthesis implantation, the specimens were placed on the workbench of a MODEL-55100 Electronic Universal Testing Machine (Changchun Testing Machine Institute, Changchun, China). The specimens were pretreated, as described by Liu et al. [12]. Subsequently, the lead lines of the electric resistance strain gauges were affixed to the femur specimens and connected to the bridge arm of the junction box. A half-bridge bridging mode was used, and the temperature compensation was external. A compression load of 0.8 kN (average weight of young males in northern China) was applied using the universal testing machine at an experimental velocity of 2 mm/min (static load), and the strain values at various measurement points were measured using a yj-4501A model static resistance strain gauge (Nanjing University of Aeronautics and Astronautics, Nanjing, China).

### Femur implantation methods for the traditional and anatomic femoral prosthesis groups

After strain electric measurements of the normal femoral specimens in the traditional and anatomic prosthesis groups, the specimens were frozen at −22°C for 24 h and then thawed at room temperature. The prostheses were implanted into the normal femoral specimens of both groups. The method for implanting the prostheses into the femurs of the traditional group was described by Liu et al. [12]. The implantations were performed by the same, highly qualified surgeon. The femoral joint was fixed on to the platform. The osteotomy was performed, 1.5 cm from the top of the small trochanter to the bottom of a large trochanter, using a clinical saw to remove the femoral head and most of the neck. Cancellous bone excavation was then performed within the distal femur to determine the location of the marrow cavity. The marrow cavity was expanded using a clinical reamer to reach the distal femur marrow cavity, along the great trochanter, and any internal debris was removed. The appropriate prosthesis was selected for biological fixation of the femoral neck to maintain an incline of 10°–15°.

The method for implanting the prostheses into the femurs of the anatomic prosthesis group was performed according to the method of Liu et al. [12]. The femurs were fixed on the operating platform, and the osteotomy was performed, vertically, to the femoral neck, under the femoral head. The outside line of the osteotomy was often 1.5 cm away from the great trochanter, and removed the femoral head. The aperture in the center of the large trochanter was opened, and the marrow cavity was expanded using a clinical file to obtain the proper thickness and curvature. The prosthesis was positioned, tightly fixing the prosthesis neck collar to the neck cortex.

### Strain gauge adhesion and measurement methods for both prosthesis groups

The strain values of each measuring point in the two groups were obtained by affixing the strain gauge to the pre-transplant normal femur specimens in each group after transplantation. The strain electrical measurements were obtained for the normal femurs in the traditional and anatomic prosthesis implantation groups.

### Stress values and stress shielding rate calculation method

Under compression loading, the femurs mainly underwent pressure and bending. The stress analysis showed that both the pressure and bending acted on the femur axis. Therefore, the resistance strain gauge was adhered to the femur axis to measure the strain stress values at each measuring point, and calculations were performed using Hooke’s law [13] (*σ* = *E* ⋅ *ε*, where, *σ* represents stress, and *ε* represents strain; the E-modulus for elasticity, is also called the macroscopic Young’s modulus). The stress values were calculated according to the elastic modulus of 17.6 GP, measured during the preliminary testing prior to this study. The implanted femurs in both prosthesis implantation groups were measured according to the stress shielding rate formula (*η* = 1 − *σ*/*σ*_0_ × 100 %, where *η* represents the stress shielding rate, and *σ* and *σ*_0_ are the stress values for the femurs in the same position before and after implantation of the normal femurs) [14].

### Statistical analysis

Measurement data were expressed as means ± SD. Data analyses were performed using SPSS, version 16.0, software (SPSS, Chicago, IL, USA). The differences between the data, from the two groups, were compared using single factor analysis of variance and the Scheffe law; *p* < 0.05 was considered to indicate statistical significance.

## Results

The results of the stress measurements at the different test points before and after traditional and anatomic femoral prosthesis implantations are shown in Table 1. The stress shielding rates are shown in Table 2. According to the differences in geometry and elastic modulus between the anatomical and traditional prostheses, the stress shielding rate of the anatomical prosthesis was lower than that of the traditional prosthesis when implanted into the femur specimens. Femoral electric strain measurements of the normal control group, the traditional prosthesis implantation group, and the anatomical prosthesis implantation group are presented (Figure 3A,B,C). Strain measurement was performed using electronic universal testing machine and imaged by resistance strain gauges (Figure 3D).

**Table 1:** **Strain electric measurements of each group (values are represented in mean (SD)) (*n*** **= 8)**

**Point**	**Normal femur after traditional prosthesis transplantation**		**Femur after traditional prosthesis transplantation**		**Normal femur after anatomic prosthesis transplantation**		**Femur after anatomic prosthesis transplantation**	
	**Strain (×10**^**−6**^**)**	**Stress (MPa)**	**Strain (×10**^**−6**^**)**	**Stress (MPa)**	**Strain (×10**^**−6**^**)**	**Stress (MPa)**	**Strain (×10**^**−6**^**)**	**Stress (MPa)**
1	−746 (21.2)	−13.1 (1.6)			−738 (23.2)	−12.9 (1.8)	−598 (31.2)	−10.5 (1.6)
2	−712 (19.4)	12.5 (1.2)			−725 (27.1)	−12.7 (1.6)	−571 (26.3)	−10.0 (1.4)
3	−1,872 (31.3)	−32.9 (2.4)			−1,894 (31.4)	−33.3 (2.7)	−1,418 (35.0)	−25.0 (1.7)
4	−2,001 (27.1)	−35.2 (1.9)			−2,016 (36.2)	−35.4 (2.7)	−1,627 (29.2)	−28.6 (2.0)
5	−1,079 (28.2)	−19.0 (1.3)			−1,098 (28.1)	−19.3 (1.8)	−816 (31.2)	−14.3 (1.4)
6	−728 (31.4)	−12.8 (1.7)	−462 (−8.13)	8.13 (0.8)	−719 (22.3)	−12.7 (1.4)	−518 (24.6)	−9.1 (0.8)
7	1,472 (29.6)	−25.0 (2.4)	798 (14.1)	14.1 (1.1)	1,416 (28.4)	24.9 (2.3)	973 (29.1)	17.1 (1.2)
8	−1,118 (27.4)	−19.7 (1.8)	−748 (19.6)	13.2 (0.8)	−1,127 (25.6)	−19.8 (1.7)	−835 (23.6)	−14.6 (1.3)
9	878 (19.6)	−15.5 (1.4)	543 (27.4)	9.6 (1.2)	864 (27.2)	15.2 (1.1)	632 (19.1)	11.1 (0.7)
10	−608 (19.8)	−10.7 (1.2)	573 (27.4)	10.1 (0.9)	−612 (19.1)	−10.8 (1.2)	593 (25.8)	−10.4 (1.2)
11	−1,563 (30.3)	−27.5 (2.8)	−1,009 (28.1)	17.7 (1.3)	−1.572 (30.2)	−27.7 (2.1)	−1,218 (28.2)	−21.4 (1.9)
12	−758 (19.6)	−13.3 (1.1)	−532 (19.9)	9.4 (0.8)	−774 (19.2)	−13.6 (1.6)	−631 (22.6)	−11.1 (1.0)
13	1,352 (27.1)	−23.8 (1.9)	962 (22.6)	15.2 (0.9)	1,364 (31.1)	24.6 (2.3)	1,139 (20.4)	20.0 (1.4)
14	−701 (28.4)	−12.3 (1.4)	−538 (18.1)	9.5 (0.6)	−717 (22.5)	−12.6 (1.4)	−608 (26.3)	−10.7 (0.9)
15	749 (18.6)	−13.2 (0.91)	562 (26.3)	9.9 (0.7)	768 (19.2)	13.5 (1.2)	627 (19.4)	11.0 (1.0)
16	−412 (28.7)	−0.73 (0.09)	371 (16.2)	65.2 (3.6)	−406 (18.1)	−7.1 (0.8)	−398 (22.4)	−7.0 (0.6)
17	−1,968 (23.1)	−34.6 (2.6)	−1,618 (31.7)	28.4 (1.6)	−1,992 (24.1)	−35.0 (2.6)	−1,789 (32.4)	−31.4 (2.6)
18	1,218 (25.1)	21.4 (1.8)	1,064 (28.2)	18.7 (1.2)	1,214 (22.4)	21.3 (2.3)	−1,163 (33.1)	20.4 (1.2)

The strain values of 6, 7, 9, 10, 11, 12, 14, 15, 16, and 18 measurement points in the reservation and traditional femoral prosthesis groups were analyzed by paired *t* test; the difference was significant (*p* < 0.05). 1, 2, 3, 4, and 5 traditional measuring points were destroyed and cannot be measured. Experimental results showed that the strain values of 6, 7, 9, 10, 11, 12, 14, 15, 16, and 18 measurement points in the traditional prosthesis group were less than those of the Germany femoral neck retention prosthesis group (*p* < 0.05).

**Table 2:** **Stress shielding rate calculations (values are represented in mean (SD)) (*n*** **= 8)**

**Point**	**Stress shielding rate in the traditional prosthesis group (*η*%)**	**Stress shielding rate in the anatomic prosthesis group (*η*%)**
1		18.6 (1.6)
2		21.2 (2.1)
3		24.9 (1.9)
4		19.2 (1.7)
5		25.4 (2.0)
6	36.5 (1.8)	28.3 (2.4)
7	43.6 (2.6)	31.4 (2.8)
8	33.0 (2.9)	26.3 (2.4)
9	38.0 (3.1)	27.0 (1.2)
10	5.6 (0.8)	3.7 (0.4)
11	35.6 (2.3)	22.7 (1.8)
12	29.3 (2.6)	18.4 (1.9)
13	36.1 (2.9)	18.7 (1.4)
14	22.7 (1.8)	15.1 (1.7)
15	25.0 (1.6)	18.5 (1.2)
16	1.5 (0.2)	1.41 (0.1)
17	17.9 (1.9)	10.3 (0.8)
18	12.6 (1.1)	4.3 (0.3)

The strain values of 6, 7, 9, 10, 11, 12, 14, 15, 16, and 18 measurement points in the reservation and traditional femoral prosthesis groups were analyzed by paired *t* test; the difference was significant (*p* < 0.05). 1, 2, 3, 4, and 5 traditional measuring points were destroyed and cannot be measured. Experimental results showed that the strain values of 6, 7, 9, 10, 11, 12, 14, 15, 16, and 18 measurement points in the traditional prosthesis group were less than those of the Germany femoral neck retention prosthesis group (*p* < 0.05).

The study aims to compare the stress shielding effects of implantable anatomical and traditional prostheses after *in vitro* total hip joint replacement simulation. The study serves as a biomechanical basis for novel artificial prostheses and for clinical hip joint replacements.

## Discussion

The study aims to compare the stress shielding effects of implantable anatomical and traditional prostheses after in vitro total hip joint replacement simulation. Compared to the traditional prosthesis group, there was a lower stress shielding rate in the proximal 1–10 stations in the femoral neck dissection-type prosthetic femur specimens in the anatomical femoral prosthesis group (*p* < 0.05). In the anatomical model, the stress shielding rate, under a load, was lower than the rate in the traditional prosthesis group. The prosthesis, inserted into the femoral cavity, changed the normal stress distribution of the proximal femur. An analysis suggested that titanium was used in the anatomical femoral prosthesis, whereas a cobalt-chromium-molybdenum alloy was used in the traditional prosthesis. Because the elastic modulus of titanium is less than that of the cobalt-chromium-molybdenum alloy, the reserved anatomic femoral prosthesis material had a role in reducing the stress shielding effect. The reason for the lower stress shielding rate, after implantation of the anatomic femoral prosthesis into the femur specimens, compared to traditional femoral implants, was the full preservation of the femoral trochanter and neck in the anatomic prosthesis specimens. Cancellous bone plays an important physiological role in loading and cushioning. Therefore, the reduced loss of cancellous bone resulted in a smaller reduction of the bone’s function to reduce loading and provide cushioning. The traditional prosthesis retained only part of the neck. Performing the osteotomy 1–1.5 cm above the small trochanter seriously undermined the metaphyseal cancellous bone system, changing the balance of power among the systems and the stress distribution at the upper end of the normal femurs. Once prosthesis is inserted femoral intramedullary, normal stress distribution of proximal femur will be changed. Stress will be spread to distal femur via intramedullary prosthesis despite the stress should be supported by proximal femur, which leads to stress shielding in proximal femur [15],[16]. This resulted in the stress shielding rate of the anatomic femoral prosthesis implantation group being lower than that of the traditional femoral prosthesis implantation group, and suggested that a proximal, geometric design of the prosthesis, with a low elastic modulus, was essential. An unreasonable design would be to increase the chance of loosening the proximal. The biomechanical effects of the prosthetic materials were also critical. Prosthesis rigidity was related to the elastic modulus and to the cross-sectional area and shapes of the materials, but the stress shielding effect of the prosthesis stem, being bulky and rigid, was not necessarily strong because the stress shielding was a result of many factors working together. The relationship of degree of matching of the proximal end was closer. Only an elastic material with an elastic modulus closer to cortical bone could reduce the stress shielding effect, and prosthesis with different materials and shapes lead to different effects of stress shielding [17]. The high elastic modulus of prosthetic material resulted in serious stress shielding following femoral transplantation; therefore, a low elastic modulus material is a better choice for prostheses.

Kim et al. [18] compared an anatomic femoral prosthesis with common biological fixation and found that the proximal anatomic prosthesis was a better match to the canal anatomy, whereas the distal prosthesis was better than biological fixation. Thus, the proximal segment had better stress conduction. The finer, remote structure not only avoided excessive flexion stiffness but also contacted the distal cortical bone, thereby reducing the stress shielding rate. Liu et al. [12] simulated total hip arthroplasty and studied the viscoelasticity of the femur and the prosthesis by inserting the retained prosthetic femoral neck-type prosthesis (from Germany) and a traditional prosthesis (from Beijing Prussia Iron and Steel Research Implants, Beijing, China). They found that the traditional prosthesis group, by removing the femoral head and femoral neck, largely damaged the femoral longitudinal interface of the bone marrow, allowing bone moisture to be lost. The collagen and elastic fibers were largely damaged, which led to reduced 7,200-s rising displacement quantity and a change in the inherent rheological characteristics. The 7,200-s rising displacement quantity was more than that in the traditional prosthesis group, because the femur neck was retained with less damage to the intramedullary canal. According to these findings, they concluded that the retained femoral neck-type of prosthesis design, from Germany, conformed to biomechanical principles. In the present study, the parameters or methods, including the simulated total hip arthroplasty, used two kinds of prosthesis, but the prosthesis implantation methods, test specimen stress, and the applied load were similar to those used by Liu et al. [12].

Although the experiment was carried out differently than those of [18] and [12], the results showed that femoral prosthesis implantation played a role in reducing stress shielding under compressive loads, consistent with a previous report [12]. This indicates that stress distribution in the proximal femur, after anatomic femoral prosthesis implantation, changed less than during traditional femoral prosthesis replacement. Thus, the femoral stress shielding rate, under compression loads, was reduced. The experimental results supported previous reports [18] suggesting that closer matching of the anatomic state of the proximal anatomic prosthesis to the medullary cavity and proximal segment matching resulted in good conduction of stress to reduce the stress shielding rate.

Walker et al. [19] reported that the femur stress level was closer to normal bone after bone cement prosthesis replacement. For uncemented prosthesis, the femoral moment of the compressive strain is 56% of the normal value but only 30% of that for cemented prostheses. The compressive strain and rod-end strain in an uncemented prosthesis are 135% and 109% of the normal values, respectively, whereas they are 151% and 115%, respectively, of the cemented prostheses. Herein, the study supports a biological fixation method for the artificial prostheses. Engh et al. [20] performed a quantitative analysis of femoral bone resorption caused by stress sheltering. They found that bone mineral density, on the fixed prosthesis side, was significantly lower than that on the contralateral side and that bone loss was most obvious in the metaphyseal area. On average, 45% of osteoporosis patients, after receiving an artificial prosthesis, showed increased stress shielding in the greater trochanter of the femur and the femoral moment area and an increased concentration of stress in the rod-end area than before replacement. The findings in the study suggested that the region of most stress shielding was the proximal femur. Therefore, this region should receive careful attention during the selection and design of artificial prosthesis and prior to the surgical placement of an artificial prosthesis.

Under the premise of achieving stability, the design of artificial prostheses should involve the selection of low-modulus elastic fibers that are resistant to friction and that can be designed into artificial joints that can be placed into medullary cavity, causing the least damage to the femur. The consistency of the angle after placement of the designed artificial joints and the physiological angle of hip joint, matching the artificial prosthesis with the femur, can avoid looseness and improve the effect.

The present experiment was conducted *in vitro*, which may influence the experimental results and be different from the situation created during a clinical hip replacement. The advantages of strain electrical technique in an *in vitro* experiment and an *in vivo* animal experiment are that they can indicate the characteristics of field measurements, and the determinations can be directly performed during internal fixation operations. The electrometric method only measures point-by-point strain on the surface of the specimen, but does not measure the full stress distribution in a specimen. The main reason is that certain areas in the strain gauge wire grid lead to limitations in understanding stress distribution in specimens and only measure the average strain in the area. The experimental data show some discreteness because of the limited number of samples and the inherent differences between biological materials. Due to the unevenness of some parts of the upper femur specimens, it was difficult to adhere the strain gauges to allow the measurement of strain in the three directions and compute the angle of the principal stress. These types of measurements should be considered in future in-depth studies.

Taken together, different effects of stress shielding can be found between the anatomical and traditional prostheses. Retained femoral anatomical implants can increase the stability of anatomical prosthesis implants and reduce stress shielding.

## Conclusions

The anatomical and traditional prostheses had different stress shielding effects. The effect of the femoral neck-preserved anatomical prosthesis implant was significant.

## Competing interests

The authors declare that they have no competing interests.

## Authors’ contributions

DW designed the study. ML performed the study. HM analyzed the data. CP wrote the paper. All authors read and approved the final manuscript.

## References

[B1] Luo ZP Dai M The research on the influence of total hip prosthesis design and installation on joint movement Orthop J China 2005 13 778 781

[B2] Dan D Germann D Burki H Hausner P Kappeler U Meyer RP Klaghofer R Stoll T Bone loss after total hip arthroplasty Rheumatol Int 2006 26 792 798 10.1007/s00296-005-0077-0 16763871

[B3] Mújica Mota RE Cost-effectiveness analysis of early versus late total hip replacement in Italy Value Health 2013 16 267 279 10.1016/j.jval.2012.10.020 23538178

[B4] Liaw CK Wu TY Hou SM Yang RS Shih KS Fuh CS Computerized ellipse method for measuring acetabular version after total hip replacement—a precision study using synthetic and real radiographs Comput Aided Surg 2013 18 195 200 10.3109/10929088.2013.779749 23528151

[B5] Randelli F Banci L Ragone V Pavesi M Randelli G Effectiveness of fibrin sealant after cementless total hip replacement: a double-blind randomized controlled trial Int J Immunopathol Pharmacol 2013 26 189 197 23527721 10.1177/039463201302600118

[B6] Keurentjes JC, Blane D, Bartley M, Keurentjes JJ, Fiocco M, Nelissen RG: **Socio-economic position has no effect on improvement in health-related quality of life and patient satisfaction in total hip and knee replacement: a cohort study.** *PLoS One* 2013, **8:**e56785. 10.1371/journal.pone.0056785 PMC3592876 23520456

[B7] McNamara BP Cristofolini L Toni A Taylor D Relationship between bone-prosthesis bonding and load transfer in total hip reconstruction J Biomech 1997 30 621 630 10.1016/S0021-9290(97)00003-1 9165396

[B8] Sangiorgio SN Ebramzadeh E Longjohn DB Dorr LD Effects of dorsal flanges on fixation of cemented total hip replacement femoral stem J Bone Joint Surg Am 2004 86 813 820 10.1302/0301-620X.86B6.14708 15069149

[B9] Fouad H In vitro evaluation of stiffness graded artificial hip joint femur head in terms of joint stresses distributions and dimensions: finite element study J Mater Sci Mater Med 2011 22 1589 1598 10.1007/s10856-011-4319-2 21505827

[B10] Beulah P Sivarasu S Mathew L Design optimization of skeletal hip implant cross-sections using finite-element analysis J Long Term Eff Med Implants 2009 19 271 278 10.1615/JLongTermEffMedImplants.v19.i4.40 21083533

[B11] Davis ET Olsen M Zdero R Papini M Waddell JP Schemitsch EH A biomechanical and finite element analysis of femoral neck notching during hip resurfacing J Biomech Eng 2009 131 10021 10028 10.1115/1.3072889 19275431

[B12] Liu GY Zhang Q Jin Y Stress and strain analysis on the anastomosis site sutured with either epineurial or perineurial sutures after simulation of sciatic nerve injury Neural Regeneration Research 2012 7 29 2299 2304 10.3969/j.issn.1673-5374.2012.29.009 PMC4268732 25538753

[B13] Liu GY Jin Y Li P Investigation of creep mechanical characteristics of femoral prostheses by simulated hip replacement Exp Ther Med 2013 5 1189 1193 23596489 10.3892/etm.2013.966 PMC3627449

[B14] Li YF Wang YJ Du WM Stress difference on femur after total hip replacement J Med Biomech 1996 11 140 143

[B15] Richmond BJ, Bauer TW, Stulberg BN: **Boner mineral density in patients undergoing uncemented THA.** *Calcif Tissure Int* 1990, **46:**145.

[B16] Sumner DR Galante JO Determinants of stress shielding: design versus materials versus interface Clin Orthop 1992 274 202 206 1729005

[B17] Harris WH Will stress shielding limit the longevity of cemented femoral components of total hip replacement? Clin Orthop 1992 247 120 125 1728997

[B18] Kim YH Kim JS Cho SH Strain distribution in the proximal human femur. An in vitro comparison in the intact femur and after insertion of reference and experimental femoral stems J Bone Joint Surg Br 2001 83 295 301 10.1302/0301-620X.83B2.10108 11284584

[B19] Walker PS Schneeweis D Murphy S Strains and micro motions of press-fit femoral stem prostheses J Biomech 1987 20 693 702 10.1016/0021-9290(87)90035-2 3654667

[B20] Engh CA McGovern TF Bobyn JD A quantitative evaluation of per prosthetic bone remodeling after cement less total hip arthroplasty J Bone Joint Surg Am 1992 74 1009 1020 1522088

